# The Chemical Compatibility and Adhesion of Energetic Materials with Several Polymers and Binders: An Experimental Study

**DOI:** 10.3390/polym10121396

**Published:** 2018-12-16

**Authors:** Trung Toan Nguyen, Duc Nhan Phan, Duy Chinh Nguyen, Van Thom Do, Long Giang Bach

**Affiliations:** 1 Faculty of Special Equipments, Le Quy Don Technical University, 236 Hoang Quoc Viet, Hanoi 100000, Vietnam; trungtoanktqs@mta.edu.vn (T.T.N.), pdnhan912@yahoo.com (D.N.P.); 2 NTT Hi-Tech Institute, Nguyen Tat Thanh University, Ho Chi Minh City 700000, Vietnam; ndchinh@ntt.edu.vn; 3 Department of Mechanics, Le Quy Don Technical University, 236 Hoang Quoc Viet, Hanoi 100000, Vietnam; 4 Faculty of Chemical Engineering and Food Technology, Nguyen Tat Thanh University, Ho Chi Minh City 700000, Vietnam

**Keywords:** adhesion, chemical compatibility, polymer-bonded explosive, surface parameters.

## Abstract

The chemical compatibility and the adhesion of energetic materials and additive materials exert a strong influence on the sensitivity, safety and performance of a polymer-bonded explosive (PBX). In this study, the chemical compatibility of hexahydro-1,3,5-trinitro-1,3,5-triazine (RDX), pentaerythritol tetranitrate (PETN) with several polymers were evaluated using the vacuum stability test (VST) and the differential scanning calorimetry (DSC); while the adhesion between RDX or PETN and each binder based on these polymers was determined through interfacial characteristics using contact angle measurement. The experimental results demonstrate that RDX and PETN are compatible with polystyrene (PS), nitrocellulose (NC) and fluoroelastomer (FKM) according to the STANAG 4147. Therefore the two polymers can be used as adhesives in PBX composition. Moreover, based on interfacial characteristics such as interfacial tension and work of adhesion, the adhesion between RDX and each binder was predicted to be better than that of PETN.

## 1. Introduction

Hexahydro-1,3,5-trinitro-1,3,5-triazine (RDX) and pentaerythritol tetranitrate (PETN) are two of the most widely used explosives in military and civilian applications because of its high energy density properties. However, RDX and PETN are highly sensitive to mechanical pulses, thus posing considerable explosion risks and difficulties during its production, transportation, storage and charge loading. To overcome these issues, RDX and PETN are commonly mixed with polymeric binders to form polymer-bonded explosive (PBX). A PBX is an explosive material consisting of high explosives, binders (including polymers and plasticizers) and other additives [1,2,3]. In PBX composition, the explosive crystals are bound together in a matrix by a small quantity of the binder. The binder medium of the PBX can absorb the mechanical impulse, hence reducing their sensitivity to mechanical shocks. In addition, with the presence of the binder, the PBX can be compressed more effectively than other explosives [1,4,5]. The low resistance to compression of the PBX not only facilitates charge compression but also improves safety during compression, making PBX compressible into any special shapes with low compression force. Therefore, the use of the polymeric binder to bind high explosive crystal has led to the widespread and safe use of PBX [1,6].

To be used as the polymeric binder in PBX composition, the polymer must possess high chemical compatibility and adhesion with explosives [7,8]. The incompatibility reaction between the explosive and the polymer may lead to accelerated aging, reduced thermal stability and in turn, accidental explosions due to decomposition reactions [7,9,10]. In addition, the low adhesion between the explosive crystals and the binder can adversely affect the sensitivity and performance of the PBX. Gaps, which are spaces between the explosive surface and the binder, can act as initiation agent (i.e., “hot spot”) if they are adiabatically compressed by an impact or a shockwave [3,8,11]. In order to achieve better adhesive capability, surface characteristics and interfacial parameters of explosives and binders must be determined.

This study aims to investigate the chemical compatibility and the adhesion of two explosives (i.e., RDX and PETN) with several polymers (i.e., PS, NC and FKM) and binders derived from these polymers to evaluate the possibility of using polymers in PBX composition. The chemical compatibility of RDX and PETN with several polymers was evaluated according to STANAG 4147 standard “Chemical compatibility of ammunition components with explosives (non-nuclear applications)” [12] using the vacuum stability test (VST) and differential scanning calorimetry (DSC). Surface tension values of explosives and binders were determined by contact angle measurement [13,14]. Based on these surface tension values, several interfacial parameters such as an interfacial tension and the work of adhesion were determined. Thus, an accurate prediction about the adhesive ability of the explosive surface and the binder can be made.

## 2. Materials and methods

### 2.1. Materials

RDX (i.e., Class-1 with melting temperature ≥202.5 °C) and PETN (i.e., Class-1 with melting temperature ≥139.0 °C) were imported from Korea. Polystyrene (PS) with the average molecular weight of 80,000 u was prepared in the laboratory. FKM elastomer (Vinylidene Fluoride–Hexafluoropropene Copolymer with the fluorine content of 66%, density of 1.81 g·cm^−3^) was commercially obtained from DuPont Company. Three types of nitrocellulose, including NC-3, NC-NB and NC-1 with a nitrogen content of 11.96%, 12.20% and 13.39%, respectively, were obtained from a factory in Vietnam. Dioctyl phthalate (DOP) purchased from Merck was used as a plasticizer to prepare the binder from PS and NC.

### 2.2. Experimental techniques and methods

#### 2.2.1. Chemical compatibility test

Sample preparation: The energetic material (RDX or PETN) was dissolved in acetone and polymer was dissolved in the appropriate solvent (PS was dissolved in toluene, NC was dissolved in acetone) at room temperature for 120 min. The solutions with energetic material and polymer were mixed and stirred at 500 rpm at 70 °C for 120 min to remove the solvent, then the mixtures of explosives and polymers with the mass ratio of 1:1 were obtained.

Determination of chemical compatibility: All measurements were performed according to STANAG 4147 [12] using vacuum stability test (VST) and differential scanning calorimetry (DSC). 

VST tests were conducted using a STABIL apparatus (OZM Research, Pardubice, Czech Republic) following the STANAG 4556-2A [15]. During the test, the sample vial was heated at 100 °C for 40 h under a vacuum pressure (≤0.672 kPa) and the volume of released gas was recorded using a pressure transducer connected to a computer [16], each experiment was carried out three times. Compatibility is judged by means of the volume of additional gases produced because of the contact between the two components of the mixture. The compatibility is determined with the following equation:

(1) $$V_{R}\;=\;M\;-\;\left(E\;+\;S\right)\;,\;\mathrm{mL}$$

where: *V_R_* is the volume of gas produced as a result of the reaction between the components of the test mixture; *M* is the volume of gas liberated from 2.5 g of explosive mixed with 2.5 g of the polymer (mL, at STP); *E* is the volume of gas liberated from 2.5 g of explosive (mL, at STP) and *S* is the volume of gas liberated from 2.5 g of polymer (ml, at STP). Following the standard, the mixture is determined as compatible if *V_R_* ≤ 5 mL and incompatible otherwise.

DSC analysis was carried out using a Diamond DSC (Perkin Elmer, Waltham, MA, USA). The DSC experiments were conducted at a heating rate of 5 K·min^-1^ under a dynamic nitrogen atmosphere (with a flow rate of 20 mL·min^−1^). The sample vial (sample mass about of 2.0 mg) was heated from 30 to 300 °C (for RDX, NC and mixtures based on RDX) and from 30 to 250 °C (for PETN and mixtures based on PETN). The difference in the exothermic peak temperature ($\Delta T_{P}$) of the DSC curve of single material compared to the mixture (i.e., a shift in peak temperature) was determined by the following equation:

(2) $$\Delta T_{P}\;=\;T_{P}^{S}\;-\;T_{P}^{M},\;{}^{\circ}C$$

where: $T_{P}^{S}$ and $T_{P}^{M}$ are the exothermic peak temperatures of the single system and the mixture of explosive with the polymer, respectively. The single system is the component in which its exothermic peak temperature is lower than that of another component in the mixture system [17,18,19]. According to the standard [12], if $\Delta T_{P}\;\leq \;4\;{}^{\circ}C$, the mixture is determined as compatible; if $\Delta T_{P}\;\geq \;20\;{}^{\circ}C$, the mixture is incompatible; if $\Delta T_{P}$ is between 4 and 20 °C, another method is recommended to determine the compatibility.

#### 2.2.2. Surface parameters calculation

Binders were PS and NC samples plasticized by DOP with different DOP/PS and DOP/NC weight ratios and FKM was dissolved in acetone. For contact angle measurements, RDX, PETN and FKM were deposited onto glass microscope slides with a thin layer and binders based on DOP/PS and DOP/NC were also coated to same slides [8].

Surface characteristics of the explosive and the binder were calculated by contact angle measurement. The contact angle was determined by the CAM 200 contact angle analyzer (KSV Instruments, Helsinki, Finland), each experiment was carried out three times. The contact angle data can be related to the surface tension of the material through Young’s equation [13,20]:

(3) $$\gamma_{SV}\;-\;\gamma_{SL}\;=\;\gamma_{LV}\cdot \cos\left(\theta \right)$$

where $\gamma_{SV}$, $\gamma_{SL}$ and $\gamma_{LV}$ are the solid-vapor, solid-liquid and liquid-vapor interfacial tensions, respectively and θ is Young’s contact angle. While the liquid-vapor surface tension $\gamma_{LV}$ and the contact angle θ are determined directly, the solid-liquid surface tension $\gamma_{SL}$ can be measured by Equation (3). In addition, the surface tension is assumed to comprise two components [8,13,21]:

(4) $$\gamma \;=\text{​}\;\gamma^{D}\;+\;\gamma^{P}$$

where $\gamma^{D}$ and $\gamma^{P}$ are the dispersive and polar components of the surface tension, respectively. The relationship of the dispersion and polar interactions between liquids and solids are described as follows [8,13,22]:

(5) $$\gamma_{LV}\;=\;\gamma_{LV}^{D}\;+\;\gamma_{LV}^{P}\;=\;\alpha_{L}^{2}\;+\;\beta_{L}^{2}$$

(6) $$\gamma_{SV}\;=\;\gamma_{SV}^{D}\;+\;\gamma_{SV}^{P}\;=\;\alpha_{S}^{2}\;+\;\beta_{S}^{2}$$

(7) $$W_{a}\;=\;2\left(\alpha_{L}\alpha_{S}\;+\;\beta_{L}\beta_{S}\right)\;=\;\gamma_{LV}[1+\cos\left(\theta \right)]$$

Equation (7) can be expressed as Equation (8):

(8) $$\frac{W_{a}}{2\alpha_{L}}\;\;=\;\;\alpha_{S}\;+\;\beta_{S}\frac{\beta_{L}}{\alpha_{L}}$$

where *W_a_* is work of adhesion; α and β are square-rooted $\gamma^{D}$ and square-rooted $\gamma^{P}$, respectively. From the Equation (7), the values *α_S_* and *β_S_* are calculated from the straight line when plotting ($W_{a}/2\alpha_{L}$) versus ($\beta_{L}/\alpha_{L}$).

The values of interfacial tension between explosive and the binder ($\gamma_{12}$) are calculated by [8]:

(9) $$\gamma_{12}\;=\;\gamma_{1}\;+\;\gamma_{2}\;-\;2\left(\alpha_{1}\alpha_{2}\;+\;\beta_{1}\beta_{2}\right)$$

where subscript 1 and 2 refer to phase 1 and 2, respectively. 

#### 2.2.3. Determination of the Resistance to Compression of PBX

PBXs of different formulations were prepared from a mixture of explosive (RDX or PETN) with the binder (DOP/PS, DOP/NC-NB—Section 2.2.2). RDX and PETN have the same particle size range of 100 to 150 µm. During the preparation of PBX, RDX or PETN was mixed with the binder in the beaker for 30 min. The obtained mixture was subsequently dried at 90 to 95 °C for 5 h. These compositions of PBXs are provided in Table 1.

The resistance of compression of PBX can be assessed by determining the shape of the explosive block with low compression pressures. PBX sample (15.0 g) was compressed into a cylinder mold (with a diameter of 24.5 mm) and then the density of the PBX block was determined. For PBX samples with the same explosive content, the greater the density, the lower the resistance of compression (i.e., the better the adhesion).

## 3. Results and discussion

### 3.1. The Chemical Compatibility

#### 3.1.1. VST Results

The extra volume of gas production *V_R_* calculated from the VST measurements following Equation (1) for all mixtures (based on RDX and PETN) was given in Table 2.

From Table 2, it showed that two mixtures of PETN and RDX with NC-1 presented the largest increase in the volume of released gas, indicating that NC-1 induces higher released gas compared to the rest of polymers. However, this is not necessarily the indication of incompatibility since the STANAG 4147 standard allows a maximum variation of 5.0 mL (STP) when materials are mixed and tested with the VST as a criterion for compatibility [7,12]. The *V_R_* values calculated for all mixtures of RDX and PETN with PS, FKM and three types of NC were lower than 1.50 mL, indicating that mixtures of these explosives and polymers are considered chemically compatible according to the STANAG 4147.

#### 3.1.2. DSC results

**Figure 1:**
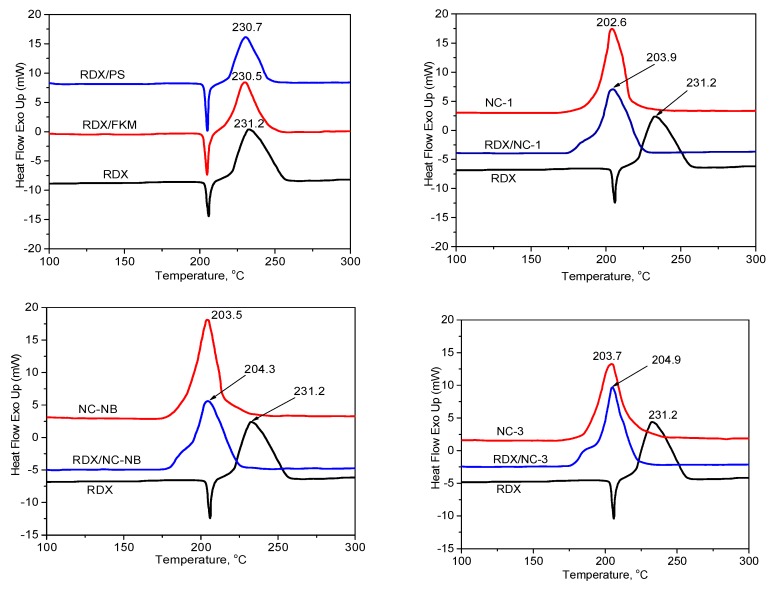
DSC curves of RDX and mixtures based on RDX.

**Figure 2:**
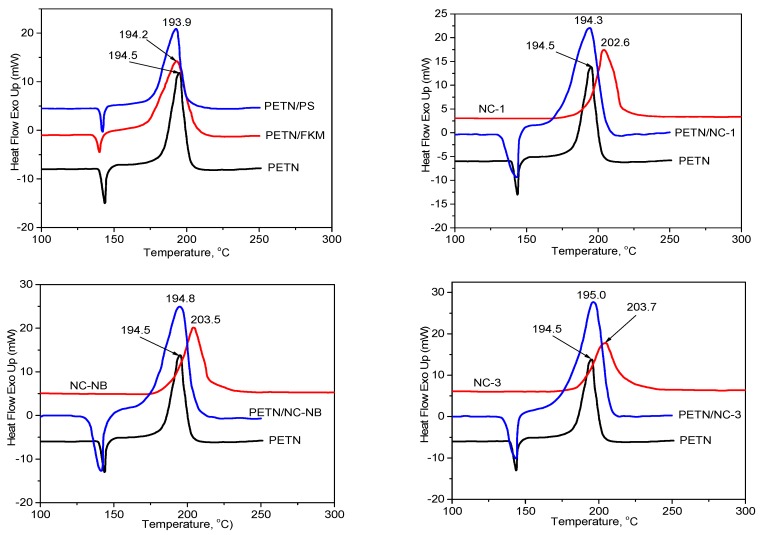
DSC curves of PETN and mixtures based on PETN.

DSC curves of explosives, polymers and mixtures are shown in Figure 1 and Figure 2 and the shift of exothermic peak temperatures are presented in Table 3. In the study of temperature ranges, the DSC curves of FKM and PS showed no exothermic peak. It is noteworthy that the maximum exothermic peak temperature of the NC sample was lower than that of the RDX sample, so NC samples were selected as the single system in the mixture system based on RDX and NC.

If the shift of the exothermic peak temperature is negative (i.e., the mixture has higher temperature decomposition than that of single energetic material), the mixture is compatible. If the shift of the exothermic peak temperature is positive, this indicates that the presence of the binder has accelerated the decomposition of the energetic materials and the compatibility of the mixture would be evaluated according to the STANAG 4147 (Section 2.2.1) [12]. From Figure 1 and Figure 2 and Table 2, the following observation can be made:

The DSC curves of RDX/NC-1, RDX/NC-NB, RDX/NC-3 and three types of NC showed only an exothermic peak observed around 203 °C relating to the decomposition of NC. The values of (∆*T_p_*) between NCs and RDX/NC-1, RDX/NC-NB, RDX/NC-3 are −1.3, −0.8 and −1.2 °C, respectively, showing that mixtures of RDX and three types of NC have good compatibility. 

The DSC curves of RDX, RDX/FKM, RDX/PS showed an endothermic peak observed between 200 and 205 °C, which indicated the melting of RDX [1] and an exothermic peak observed around 230 °C related to the decomposition peak of RDX. The values of (∆*T_p_*) between RDX with RDX/FKM and RDX/PS are 0.7 and 0.5 °C, respectively. According to the STANAG 4147, mixtures of RDX with PS and FKM are considered compatible.

The DSC curves of PETN and mixtures of PETN with PS, FKM and NCs showed an endothermic peak observed between 140 and 150 °C relating to the melting of PETN [1] and an exothermic around 194 °C, which indicated the decomposition peak of PETN. The values of (∆*T_p_*) between PETN with PETN/FKM, PETN/PS, PETN/NC-1, PETN/NC-NB and PETN/NC-3 are 0.3, 0.6, 0.2, −0.3 and −0.2, respectively. By STANAG 4147, mixtures of PETN and FKM, PS and NCs are considered compatible.

The compatibility results obtained from the DSC method appear to be contradicted to those obtained from the VST method. This seeming contradiction might be attributable to the different principles of these two methods. Indeed, advantages of DSC methods are the use of a small amount of sample and the short time needed for measurements. Nevertheless, a small amount of sample could be a disadvantage because of sample inhomogeneities. Meanwhile, the greatest advantage of VST in relation to DSC is the sample weight (2.5 g of each material). However, the VST has some drawbacks: the condensation, adsorption of released gas with test materials and the VST experiments take a long time. For these reasons, the result of the compatibility test using VST and DSC methods may be different. It is noteworthy that Vogelsanger [7], Haye et al. [23] and Myburgh [24] even reported completely contradictory compatibility results (compatible–incompatible) between the DSC and VST methods.

### 3.2. The Interfacial Parameters

Contact angle measurement

The contact angles of RDX and PETN with several standard solvents were determined using the CAM 200 apparatus (KSV Instruments). The surface parameters of these solvents were given in Table 4.

The results of contact angle measurement of energetic material with some binder are expressed in Table 5.

**Figure 3:**
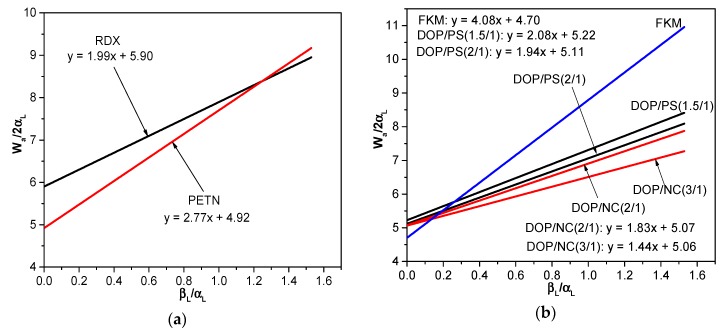
The ($W_{a}/2\alpha_{L}$) versus ($\beta_{L}/\alpha_{L}$) plot of (**a**) energetic materials and (**b**) several binders.

**Figure 4:**
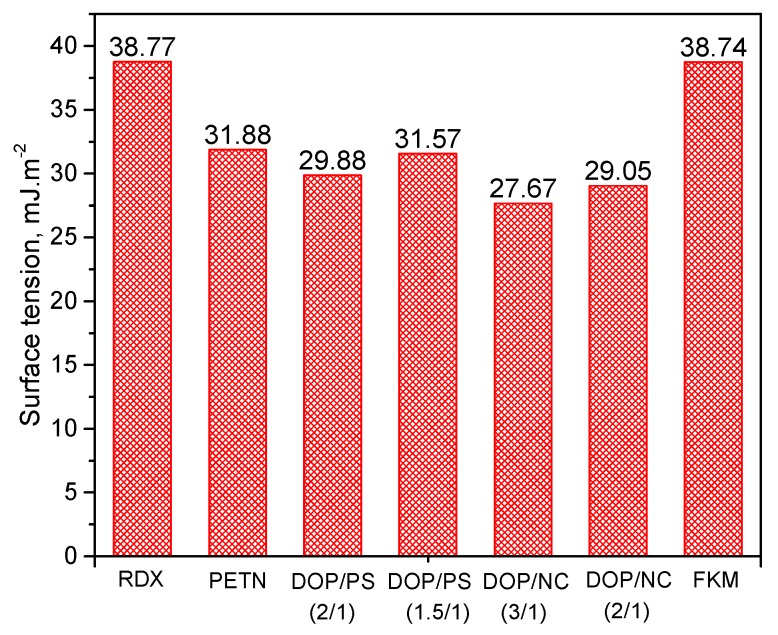
Surface tensions of energetic materials and several binders.

According to the Equation (8), the intercept $\alpha_{S}$ and the slope $\beta_{S}$ were determined by the plotting of ($W_{a}/2\alpha_{L}$) versus ($\beta_{L}/\alpha_{L}$), so that surface tension values of the energetic material and the binder could be determined. The results are shown in Figure 3 and Figure 4.

**Figure 5:**
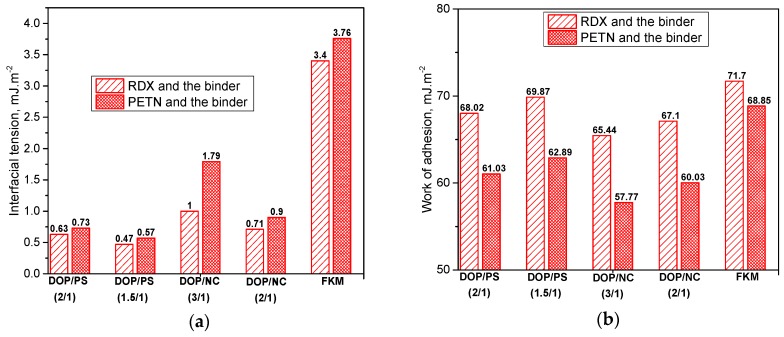
(**a**) Interfacial tension and (**b**) Work of adhesion of explosives and several binders.

From the surface tension values, the interfacial tensions and the works of adhesion between energetic materials and the binders are calculated according to the Equations (7) and (9), respectively. The results are shown in Figure 5.

**Figure 6:**
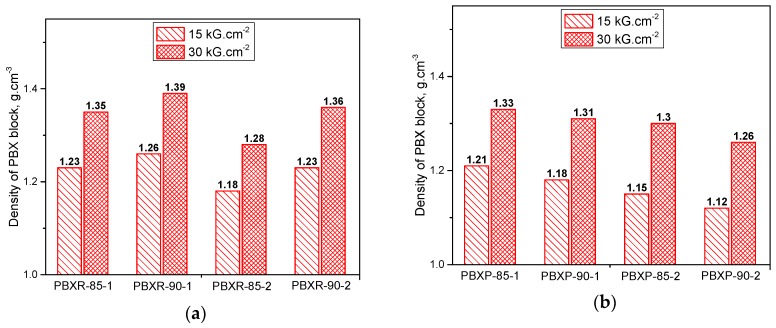
The density of PBX blocks based on RDX (**a**) and PBX blocks based on PETN (**b**) at different compression pressures.

When explosive and the binder bonded together, the smaller the value of interfacial tension, the better their adhesion. Similarly, the greater the value of work of adhesion, the better adhesion between them [8,13,22]. As shown in Figure 5, all of the mixtures of RDX and each binder have the smaller interfacial tension and the higher work of adhesion than that of PETN, so the adhesion of RDX to the binder is considered better compared to PETN. In addition, the interfacial tension and the work of adhesion results demonstrated that the adhesion of the DOP/PS based binder to explosives is better than that of the DOP/NC based binder. Particularly for the case of FKM, the rules on the suitability of the interfacial tension and the work of adhesion of explosive and FKM are unclear. The difference in adhesion of each PBX system was confirmed by the density of PBX blocks under several compression pressure (Figure 6).

As seen in Figure 6, increased RDX content in the PBXR components from 85 to 90 wt % results in their higher density (at the same compression pressure). In contrast, the density of PBXP samples (90 wt % PETN) are slightly lower compared to PBXP samples containing 85 wt % PETN (under the same compression condition). Thus, as the increase of PETN content from 85 to 90 wt %, the adhesion of PETN with binders tended to decrease, indicating that the adhesion of PETN with each binder is less than that of RDX. Moreover, densities of PBXR-85-1, PBXR-90-1, PBXP-85-1 and PBXP-90-1 samples (contain DOP/PS binder) tend to be slightly higher than those of PBXR-85-2, PBXR-90-2, PBXP-85-2 and PBXP-90-2 (contain DOP/NC binder), respectively.

## 4. Conclusions

The chemical compatibility of energetic materials and several polymers were investigated using DSC and VST analysis. Based on results of DSC and VST examinations, it is possible to conclude that all mixtures of two explosives (i.e., RDX and PETN) with several polymers including PS, NC, FKM are compatible according to the STANAG 4147. Therefore, it is possible to use these polymers as adhesives in PBX compositions based on RDX and PETN.

In addition, interfacial parameters of RDX and PETN with several binders based on PS, NC and FKM were determined using contact angle measurements. The interfacial tension between RDX and each binder is lower than that of PETN. In contrast, the values of the work of adhesion of RDX-based mixtures are higher than those of PETN. These results indicated that the adhesion of RDX and each binder is better than that of PETN. On the other hand, the adhesive ability to explosives of the binder based on PS is better than that of the binder based on NC. 

## Figures and Tables

**Table 1:** Compositions of PBX based on RDX and PETN.

Compositions	Content of Material, wt %			
	RDX	PETN	DOP/PS (2/1)	DOP/NC-NB (2/1)
PBXR-85-1	85	-	15	-
PBXR-85-2	85	-	-	15
PBXR-90-1	90	-	10	-
PBXR-90-2	90	-	-	10
PBXP-85-1	-	85	15	-
PBXP-85-2	-	85	-	15
PBXP-90-1	-	90	10	-
PBXP-90-2	-	90	-	10

**Table 2:** The volume of gas released obtained by the vacuum stability test.

Sample	*V_R_*, mL				
	PS	FKM	NC-1	NC-NB	NC-3
RDX	0.03 ± 0.01	0.09 ± 0.02	0.90 ± 0.04	0.27 ± 0.03	0.13 ± 0.02
PETN	0.05 ± 0.02	0.13 ± 0.02	1.00 ± 0.07	0.42 ± 0.04	0.20 ± 0.04

**Table 3:** The chemical compatibility investigated by DSC measurements.

Samples		The Exothermic Temperature Peak		$\Delta T_{P}$, °C
Mixture Systems (1/1)	Single System	$T_{P}^{S}$, °C	$T_{P}^{M}$, °C	
RDX/FKM	RDX	231.2	230.5	0.7
RDX/PS	RDX	231.2	230.7	0.5
RDX/NC-1	NC-1	202.6	203.9	-1.3
RDX/NC-NB	NC-NB	203.5	204.3	-0.8
RDX/NC-3	NC-3	203.7	204.9	-1.2
PETN/FKM	PETN	194.5	194.2	0.3
PETN/PS	PETN	194.5	193.9	0.6
PETN/NC-1	PETN	194.5	194.3	0.2
PETN/NC-NB	PETN	194.5	194.8	-0.3
PETN/NC-3	PETN	194.5	195.0	-0.5

**Table 4:** Surface parameters of several solvents used to measure the contact angle.

Solvent	$\gamma_{LV},\;\mathrm{mJ}\cdot m^{-2}$	$\gamma^{D}$	$\gamma^{P}$	$\alpha_{L}$	$\beta_{L}$
Water	72.8	21.8	51.0	4.67	7.14
Glycerol	63.1	37.0	26.1	6.08	5.11
Ethylene glycol	44.4	31.7	12.7	5.63	3.56
Chloroform	27.2	27.2	0	5.21	0

**Table 5:** The contact angle of energetic material and the binder.

Sample	Water	Glycerol	Ethylene Glycol	Chloroform
RDX	81.50 ± 1.91	62.51 ± 2.03	35.32 ± 1.32	Spread *
PETN	79.45 ± 1.42	69.20 ± 2.35	42.41 ± 2.74	Spread *
DOP/PS = 2/1	88.68 ± 1.10	72.49 ± 1.82	47.00 ± 1.42	26.20 ± 2.40
DOP/PS = 1.5/1	86.05 ± 2.31	70.60 ± 2.07	43.02 ± 1.54	18.32 ± 2.37
DOP/NC = 3/1	94.64 ± 1.89	79.54 ± 2.76	50.83 ± 1.52	30.36 ± 1.66
DOP/NC = 2/1	89.76 ± 1.03	76.72 ± 2.20	46.50 ± 2.09	27.23 ± 1.25
FKM	66.78 ± 2.23	50.02 ± 2.17	39.24 ± 1.98	Spread *

Spread *: the drop began to spread immediately after it was deposited on the surface.
